# The way to precision medicine in gynecologic cancers: The first case report of an exceptional response to alpelisib in a *PIK3CA*-mutated endometrial cancer

**DOI:** 10.3389/fonc.2022.1088962

**Published:** 2023-01-13

**Authors:** Anna Passarelli, Jole Ventriglia, Carmela Pisano, Sabrina Chiara Cecere, Marilena Di Napoli, Sabrina Rossetti, Rosa Tambaro, Luca Tarotto, Francesco Fiore, Alberto Farolfi, Michele Bartoletti, Sandro Pignata

**Affiliations:** ^1^ Department of Urology and Gynecology, Istituto Nazionale Tumori, Istituto di Ricovero e Cura a Carattere Scientifico (IRCCS) Fondazione G. Pascale, Naples, Italy; ^2^ Interventional Radiology Unit, Istituto Nazionale Tumori, Istituto di Ricovero e Cura a Carattere Scientifico (IRCCS) Fondazione G. Pascale, Naples, Italy; ^3^ Department of Medical Oncology, Istituto Nazionale Tumori, Istituto di Ricovero e Cura a Carattere Scientifico (IRCCS) Istituto Romagnolo per lo Studio dei Tumori Dino Amadori, Meldola, Emilia-Romagna, Italy; ^4^ Unit of Medical Oncology and Cancer Prevention, Department of Medical Oncology, Centro di Riferimento Oncologico di Aviano (CRO), Aviano, Italy

**Keywords:** alpelisib, endometrial cancer, phosphatidylinositol 3-kinase (PI3K) pathway, genomic profiling, PI3K inhibitor, PIK3CA mutation

## Abstract

Endometrial cancer (EC) is the most common gynecologic cancer in Europe and its prevalence is increasing. EC includes a biological and clinical heterogeneous group of tumors, usually classified as type I (endometrioid) or type II (non-endometrioid) based on the histopathological characteristics. In 2013, a new molecular classification was proposed by The Cancer Genome Atlas (TCGA) based on the comprehensive molecular profiling of EC. Several molecular somatic alterations have been described in development and progression of EC. Using these molecular features, EC was reclassified into four subgroups: POLE ultra-mutated, MSI hypermutated, copy-number low, and copy-number high that correlate with the prognosis. To this regard, it is widely reported that EC has more frequent mutations in the phosphatidylinositol 3-kinase (PI3K) pathway signaling than any other tumor. *PIK3CA* is the main significant mutated gene after *PTEN* alterations. Overall, over 90% of endometrioid tumors have activating PI3K molecular alterations that suggests its critical role in the EC pathogenesis. Thus, the dysregulation of PI3K pathway represents an attractive target in EC treatment. Herein, we report a radiological and clinically meaningful response to a selective PIK3 inhibitor in a patient with extensively pre-treated advanced endometrioid EC harboring a somatic activating *PIK3CA* hotspot mutation. These evidences provide the rational for translational strategies of the PI3K inhibition and could support the clinical usefulness of *PIK3CA* genotyping in advanced EC. To our knowledge, this is the first clinical case of *PIK3CA*-mutated EC successfully treated with alpelisib.

## Introduction

Endometrial cancer (EC) is the only gynecological tumor that is rising in terms of incidence and associated mortality worldwide (1). EC is typically diagnosed in the early stages when the disease is confined to the uterus. EC patients affected by early-stage disease have a good prognosis, and for all patients with EC in Europe (all disease stages) have been reported 5-years survival rates of 76% (2). Anyway, women with advanced or recurrent EC show lower response rates to the standard treatments, and clinical outcomes are extremely poor.

Therefore, the development of further therapeutic strategies for these patients is needed, hopefully based on the novel aspects of precise molecular pathogenesis of EC.

Historically, EC have been classified into two different groups (3). Type I endometrioid tumors are linked to hormone-receptor positivity, estrogen excess, obesity, and favorable prognosis compared with type II tumors that are more frequent in non-obese women, older, and have a worse outcome. In 2013, the Cancer Genome Atlas (TCGA) proposed a new classification through the evaluation of the genomic and epigenomic landscapes of primary EC. In specific, TCGA delineated four molecular entities: polymerase ϵ (POLE)-mutant/hypermutated, microsatellite instability-high (MSI-H), copy number high, and copy number low (4),. Interestingly, this new classification is related to the underlying tumor biology and promising therapeutic strategies.

In recent years several somatic mutations have been identified and related targeted therapies have shown promising success.

For example, the molecular subtype with Mismatch Repair Deficiency (dMMR)/or MSI-H occurs in 23-36% of EC and is associated with immune activation (5). Therefore, in August 2021 the Food and Drug Administration (FDA) has granted accelerated approval for the anti-PD-1 dostarlimab in recurrent dMMR/MSI EC, based on a phase I trial (6).

Interestingly, the phosphatidylinositol 3-kinase (PI3K)/mammalian target of rapamycin (mTOR) pathway is frequently dysregulated in EC, often due to activating mutations or amplification of PIK3CA (7–9). PI3K alpha is a heterodimeric protein complex comprised of the catalytic p110 alpha subunit and the regulatory p85a subunit, which are encoded by the PIK3CA and PIK3R1 genes respectively. The common mechanisms of the PI3K alpha activation in carcinogenesis are the acquisition of somatic gain-of-function mutations within PIK3CA or loss of PTEN activity. Several studies revealed that over 80% of all *PIK3CA* mutations occurred within exons 9 and 20, while only in 20% within exons 1-7.

Therefore, targeting the PI3K/mTOR pathway is an attractive strategy and may be particularly active in solid tumors that signal heavily through PI3Ka such as those harboring PIK3CA alterations.

Mutations in *PIK3CA* lead to increased activation of the PI3K/AKT/mTOR pathway, occurring in 93% of type 1 and 33% to 38% of type 2 EC (10). In addition, as reported by Stelloo and colleagues in a retrospective molecular analyses study of samples of EC (11), *PIK3CA* alterations were reported in the four molecular subtype, in specific in 23.9% of copy number high, in 33% in MSI, in 51.1% in POLE-hypermutated, and in 31.6% in copy number low subgroup.

It has been reported that the overactivation of this pathway in association to loos of PTEN function, is associated with poor survival in advanced solid tumors (12).

To this regard, alpelisib, an oral PI3K alpha-selective inhibitor, showed preliminary encouraging activity results in a selected population of advanced solid tumors harboring *PIK3CA* alterations, supporting the rationale for the use of PI3K pathway inhibition for the treatment of *PIK3CA*-mutant tumors (13).

We report a *PIK3CA*-mutated advanced endometrioid EC case extensively pre-treated that responded favorably to PI3K alpha-selective inhibitor namely alpelisib.

To our knowledge, this is the first clinical case of *PIK3CA*-mutated advanced EC successfully treated with alpelisib.

## Case presentation

In March 2022, a 51-year-old woman was referred to our institution for a *second opinion*.

Her past medical history was significant for the diagnosis, at the age of 47 years, of endometrial cancer. In April 2018, she underwent a radical hysterectomy with pelvic lymphadenectomy. Histology revealed an intermediate differentiated endometrioid EC, FIGO stage IIIC1 (pT3pN1). Estrogen and progesterone receptors were positive. Mismatch-Repair-Proteins such as mutL homolog 1 (MLH1), postmeiotic segregation increased 2 (PMS2), mutS homolog 2 (MSH2) and mutS homolog 6 (MSH6) were expressed.

The risk factors suggested the need of adjuvant chemoradiation treatment.

Following multi-disciplinary discussion, a sequential adjuvant radio-chemotherapy was proposed to the patient. She consented to a course of 4 cycles of 3 weekly Carboplatin (AUC 5) and Paclitaxel (175 mg/m^2^), which she received from May to July 2018, and external beam radiotherapy completed in November 2018. Three months after finishing the adjuvant therapy cycle a follow-up thoracic and abdominal computed tomography (CT) scan showed complete remission.

Ten months after stopping adjuvant therapy, the patient showed a radiological disease progression to the peritoneal carcinomatosis nodules. In consideration of the platinum free-interval, the patient was treated with carboplatin plus paclitaxel chemotherapy at standard dose for a total of six cycles.

After approximately 2 months, she experienced disease progression with increased of peritoneal nodules for which she was subjected to cytoreductive surgery on peritoneum. The histological examination confirmed the diagnosis of endometrioid EC with a high expression of hormone receptors, microsatellite stability.

The patient then received hormonal therapy with megestrol acetate for 9 months until new peritoneal disease progression.

Therefore, from January to April 2021, the patient underwent second-line chemotherapy with liposomal doxorubicin until disease progression.

From April to July 2021, the patient received intravenous weekly topotecan without any benefit.

In August 2021 started fourth-line of oral chemotherapy with cyclophosphamide until the progression of disease.

In November 2021, a new clinical progression associated to radiologic progression to peritoneum occurred. Thus, the patient received a fifth-line therapy with oral etoposide.

In January 2022, for a new lymph node and peritoneal progression associated to painful abdominal symptoms, the patient started hormone therapy with letrozole at the dosage of 2,5 mg daily.

**Figure 1:**
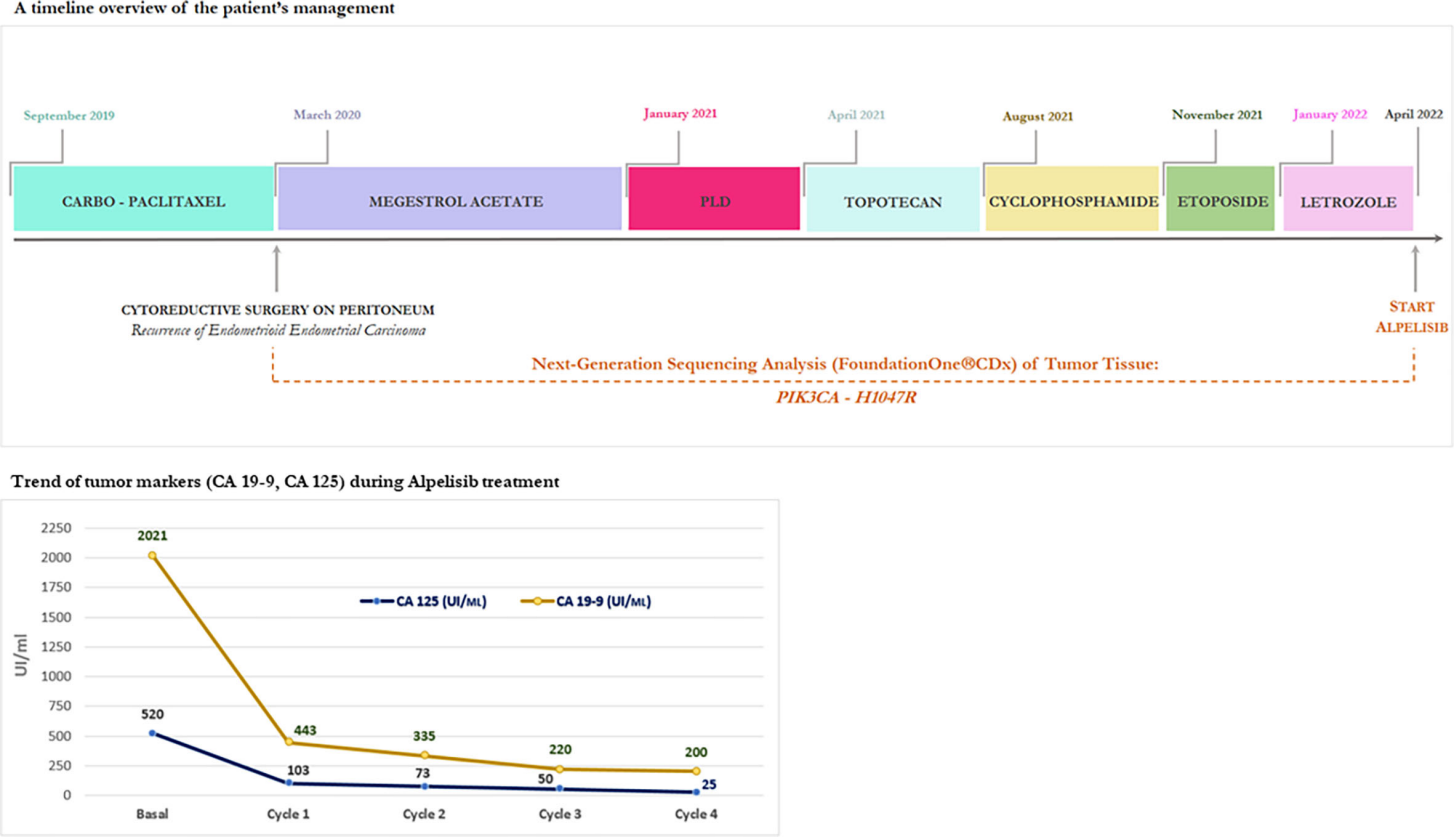
** **A timeline overview of the patient’s management and trend of tumor markers (CA 19-9, CA 125) during Alpelisib treatment.

A timeline overview of the patient’s management is summarized in **Figure 1**.

In March 2022, the patient referred to our institution in order to evaluate the potential enrollment in experimental clinical trials. Given the unavailability of clinical trials in our Institute for this type of patient, we proposed to perform a comprehensive genomic profiling through next generation sequencing (NGS) on archival tumor tissue from the last surgery. Importantly, the combination of pembrolizumab and lenvatinib was not yet available in Italy for patients with advanced EC that is not MSI-H or dMMR, who have disease progression following prior systemic therapy in any setting (14).

The patient had a personal history of hypertensive heart disease in pharmacological therapy, and iatrogenic hypothyroidism currently treated with thyroid hormone replacement. The patient was functioning well, as indicated by an Eastern Cooperative Oncology Group performance status of 1.

The NGS (*FoundationOne CDx* assay) was performed on tumor sample and showed microsatellite stability and low Tumor Mutational Burden (TMB) (1 mut/Mb) (see **Table 1**). Moreover, the data analysis revealed several genomic alterations (**Table 1**) including *PIK3CA* mutation in exon 20 (*H1047R*, variant allele frequency 78.2%), which was considered targetable.

Table 1Summary of molecular analysis – variants identified through test NGS (FoundationOne^®^CDx).

**Table:** 

GENOMIC SIGNATURES:	Result	Therapy and Clinical Trial Implications
**Microsatellite Status – MS**	MS-Stable	No therapies or clinical trials
**Tumor Mutational Burden**	1 Muts/Mb	No therapies or clinical trials

**Table:** 

GENE ALTERATIONS:	Alteration	Coding Sequence Effect	VAF (%)	Therapy and Clinical Trial Implications
**PIK3CA**	H1047R	314OA>G	78.2%	In patient’ tumor type: None. In other tumor type: Temsirolimus; Everolimus; Alpelisib.
**ARID1A**	Q507* Y1279*	1519C>T 3837T>G	37.9% 38.5%	No therapies or clinical trials No therapies or clinical trials
**CTNNB1**	S37C	110C>G	37.5%	No therapies or clinical trials
**NOTCH1**	R1594Q - subclonal	4781G>A	1.3%	Gene alteration with no reportable therapeutic or clinical trial options

**Table:** 

VARIANT OF UNKNOWN SIGNIFICANCE (VUS)			
**ATM** L2492R	ERBB2 Amplification	**KDR** R842H	**KIT** T67S
**NKKBIA** P65A	**PDGFRB** S1006A	**ROS1** G1027D and T299I	

NGS, next-generation sequencing; VAF, variant allele frequency.

In April 2022, given the lack of validated standard treatment, following discussion in the Molecular Tumor Board (MTB) of MITO for gynecological cancer patients (15), the patient started treatment with oral PI3K alpha-selective inhibitor namely alpelisib at the standard dose of 300 mg once daily on a continuous schedule in 28-day cycles until disease progression, unacceptable toxicity, or withdrawal of consent.

Following approval by the Institute’s ethics committee, alpelisib was provided for compassionate use by Novartis.

**Figure 2:**
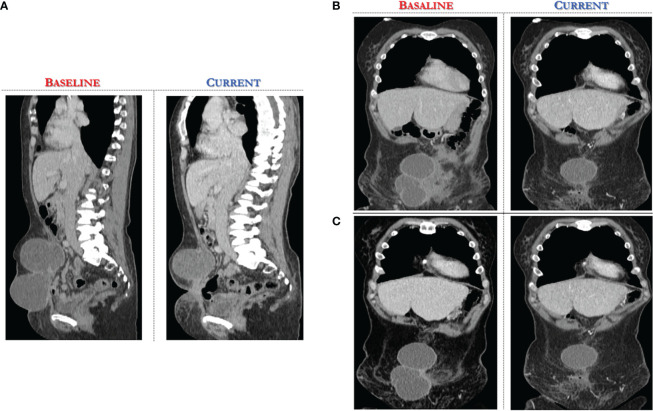
Computed Tomography scan demonstrates significant reduction of target lesions after 4 months of Alpelisib treatment compared with the corresponding pre-treatment scan. **(A)** sagittal section of CT abdomen/pelvis revealing at baseline two bulky nodules of peritoneal carcinomatosis (83 mm, 85 mm). **(B, C)** coronal view of CT abdomen/pelvis, different scans.

**Figure 3:**
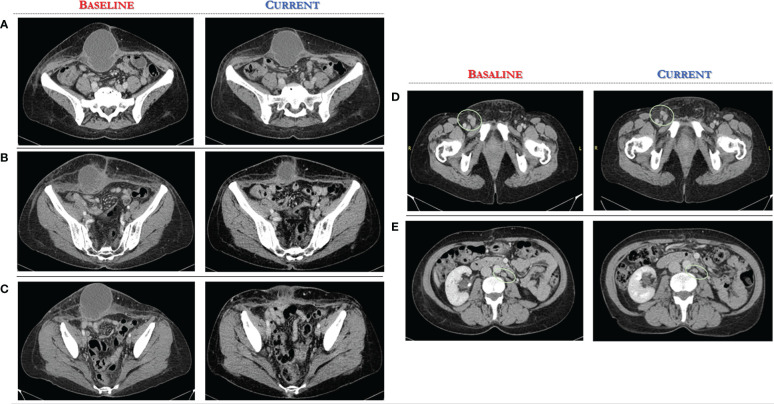
Computed Tomography scan demonstrates significant reduction of target lesions after 4 months of Alpelisib treatment compared with the corresponding pre-treatment scan. **(A-C)** axial CT scan, several sections of peritoneal carcinomatosis pre and post alpelisib treatment. **(D, E)** axial CT scan, several sections of secondary lymph nodes in the right inguinal and paraaortic region pre and post alpelisib treatment.

A CT scan at baseline revealed two bulky nodules of peritoneal carcinomatosis (83 mm, 85 mm), a 15 mm mass in the paraaortic lymph node, and a 22 mm mass in the right inguinal lymph node (see **Figures 2**, **3**).

During the course of alpelisib treatment, the patient experienced minimal toxicity, including fatigue (grade 1), and decreased appetite (grade 1), based on the CTCAE (version 5.0). Interestingly, the patient not experienced the onset of adverse events as hyperglycemia and skin reactions. To reduce the onset of skin adverse events, the patient was taking prophylactic non-sedating antihistamines such as cetirizine 10 mg once daily during the first 8 weeks of therapy then taper off.

Unfortunately, the patient developed grade 3 diarrhea (increase of ≥7 stools/day) at 30 days of alpelisib requiring treatment with loperamide and temporary interruption of alpelisib until recovery to grade ≤1. Then, the dose of alpelisib was reduced from 300 mg to 200 mg daily, which was well tolerated.

In terms of effectiveness, after just completing one 28-day cycle, she obtained a clinical benefit with meaningful improvement in her abdominal distension and discomfort, size reduction of peritoneal lesions associated to rapid and progressive reduction of tumor marker (CA 125, CA 19-9), as shown in **Figure 1**.

After only 4 months of alpelisib treatment, she achieved a partial radiologic response with complete disappearing of one of two nodules of peritoneal carcinomatosis and dimensional reduction of para-aortic and inguinal lymph nodes, as shown by the CT-scan (see **Figures 2**, **3**).

Therefore, in consideration of the objective and radiological partial response, the clinical benefit and the absence of unacceptable toxicity, by the time of writing (7 months after the start of treatment), the patient is still on therapy with alpelisib 200 mg daily.

## Discussion

In advanced EC setting, the strategy based on the use of platinum-regimens is the most active. Advanced EC patients who progress after fist line therapy have a poor prognosis and the subsequent options available are disappointing.

Given that PIK3 pathway activation is involved in the pathogenesis of EC, PIK3 pathway inhibitors are often investigated in this setting.

Here we report an emblematic case of patient with extensively pre-treated advanced endometrioid EC harboring a somatic activating *PIK3CA* mutation in exon 20 (*H1047R*), effectively treated with alpelisib as a part of a compassionate use program.

It is now known that EC harbors more frequent mutations in the PI3K/AKT pathway than any other tumor type analyzed by TCGA so far (4). *PIK3CA* and *PIK3R1* mutations were frequent and showed a strong trend for mutual exclusivity in all molecular subgroups, but they occurred with *PTEN* mutations in the MSI and copy-number low subgroups.

Previously, it has been also reported that the increased PI3K/AKT/mTOR signaling in EC is associated with aggressive phenotype disease and a poor prognosis, regardless of endometrial cancer type (16).

The PI3K pathway represents a target highly druggable and several classes of agents including rapalogs, PI3K isoform-specific inhibitors, dual PI3K/mTOR catalytic inhibitors, pan-PI3K inhibitors, mTOR-specific catalytic inhibitors, and AKT inhibitors, are in clinical development. There are several registered clinical trials of PI3K/mTOR inhibitors as a single drug or in combination for the treatment of EC.

Robust preliminary data have shown that the PI3K/AKT/mTOR pathway inhibition may be effective in patients with activating mutations in *PIK3CA* and/or loss of PTEN. In patients with breast and gynecologic malignancies, a retrospective phase I clinical trial reported significantly higher response rates in cancers with *PIK3CA* mutations (30%) compared with non-*PIK3CA* mutated tumors (10%) treated with PI3K/AKT/mTOR inhibitors as single agents or in combination with alternative therapies. The response rate was 33%, when considering only patients with EC (17). Although these data suggest *PIK3CA* mutations may have a predictive role of response, only 6 patients with EC and *PIK3CA* mutations were evaluable for response, thus further prospective clinical trials are required to conclude regarding the predictive role of *PIK3CA* mutations.

Regarding the specific function of PI3K isoform-specific inhibitors, alpelisib has demonstrated anti-cancer activity in several cancer cell lines and tumor xenograft models, in particular those harboring *PIK3CA* mutations or amplifications, underlining the enhanced clinical effectiveness in patients with PIK3CA-altered advanced solid tumors.

Our decision to manage this case with alpelisib was based on the positive preliminary NCT01219699 trial results.

In fact, in 2018 Juric and colleagues reported in the first-in-human study (NCT01219699) a favorable safety profile and encouraging signs of anti-tumor activity of alpelisib, an oral PI3K alpha-selective inhibitor, in patients with *PIK3CA*-mutant, ER-positive/HER2-negative breast cancer and other *PIK3CA*-altered advanced solid cancers (13). In this small study, the enrolled patients with diagnosis of advanced endometrial cancer were three. Interestingly, the only complete response was reported in a patient with endometrial cancer. In detail, overall response rate was 6%, stable disease was achieved in 70 patients (52.2%) and was maintained >24 weeks, disease control rate (complete and partial responses and stable disease) was 58.2%.

In addition, although the sample size was small, patients with *PIK3CA* helical domain mutations (E545K or E542K), unusual kinase mutations, or PTEN loss had partial or complete response, whereas no responses were observed in patients with kinase mutations on H1047 contrarily to our case report. PTEN mutations, which potentially may contribute to PI3K inhibitor resistance, were detected in five patients, including three whose disease progressed during the first two treatment cycles.

Since the predictive nature of these biomarkers remains not well defined, selected patients with advanced EC should be enrolled in clinical trials, and tumor samples should be collected for definition of PI3K/AKT/mTOR pathway activation status.

In gynecologic oncology, the role of predictive molecular biomarkers will continue to expand and to provide new therapeutic approaches for the management of EC patients with subsequently improvement of outcomes of efficacy and safety. To this regard, several interesting phase II basket-trials as TAPISTRY (NCT04589845) and ROME trial (NCT04591431) are ongoing and test specific targeted drugs based on molecular tumor profiling, also in pre-treated gynecologic tumors (for instance, TAPISTRY trial: *PIK3CA*➔drug Inavolisib; ROME trial: *PIK3CA*➔drug Ipatasertib) (18).

The experience in this case suggests that the precision medicine in gynecologic oncology through the detection of molecular tumor biomarkers could significantly downstage tumors also in advanced stage of disease and in extensively pre-treated patients.

Indeed, MTB can identify innovative pharmacological approaches, expanded access program or ongoing clinical trials based on tumor NGS data in patients for which there are no valid therapeutic alternatives.

Specifically, based on the effectiveness data of alpelisib in our case, we recommend referral to specialized centers the advanced EC patients and incorporation of the comprehensive NGS into care in order to offer the best chance of treatment. In consideration of the high frequency of *PIK3CA* tumor alterations in advanced EC, the genomic sequencing of the tumor tissue should be performed to evaluate for a druggable targets. To this regard, additional prospective trials are underway and needed.

## Conclusion

To our knowledge, we report for the first time a case of advanced endometrioid EC harboring a somatic *PIK3CA* mutation with an exceptional response and a good tolerance to a PI3K alpha-selective inhibitor. Our study provides unequivocal clinical evidence for the alpelisib effectiveness in treating EC cancer patients with *PIK3CA* mutation.

Future prospective studies are warranted to validate both efficacy and safety of therapy with PIK3-inhibitors in patients harboring activating *PIK3CA* mutation.

## Data availability statement

The original contributions presented in the study are included in the article/supplementary material. Further inquiries can be directed to the corresponding author.

## Ethics statement

The patient provided their written informed consent to participate in this study. Written informed consent was obtained from the individual for the publication of any potentially identifiable images or data included in this article.

## Author contributions

Experimental study design: AP and SP. Writing of the manuscript: AP and SP. Data analysis and interpretation: AP, SP and LT. Revision of the manuscript: AP, SP, LT, FF, JV, AF, MB, CP, SC, MN, RT, SR. All authors contributed to the article and approved the submitted version.
